# Genomic variant sharing: a position statement

**DOI:** 10.12688/wellcomeopenres.15090.2

**Published:** 2019-12-04

**Authors:** Caroline F. Wright, James S. Ware, Anneke M. Lucassen, Alison Hall, Anna Middleton, Nazneen Rahman, Sian Ellard, Helen V. Firth

**Affiliations:** 1 Institute of Biomedical and Clinical Science, University of Exeter, Exeter, UK; 2 National Heart and Lung Institute, Imperial Centre for Translational and Experimental Medicine, London, UK; 3 Department of Clinical Ethics and Law, Faculty of Medicine, University of Southampton, Southampton, UK; 4 PHG Foundation, Cambridge, UK; 5 Faculty of Education, University of Cambridge, Cambridge, UK; 6 Connecting Science, Wellcome Genome Campus, Cambridge, UK; 7 Division of Genetics and Epidemiology, Institute of Cancer Research, UK, London, UK; 8 Department of Clinical Genetics, University of Cambridge Addenbrooke's Hospital Cambridge, Cambridge, UK; 9 Wellcome Trust Sanger Institute, Cambridge, UK

**Keywords:** medical genomics, variant, data sharing, data ethics

## Abstract

Sharing de-identified genetic variant data via custom-built online repositories is essential for the practice of genomic medicine and is demonstrably beneficial to patients. Robust genetic diagnoses that inform medical management cannot be made accurately without reference to genetic test results from other patients, population controls and correlation with clinical context and family history. Errors in this process can result in delayed, missed or erroneous diagnoses, leading to inappropriate or missed medical interventions for the patient and their family. The benefits of sharing individual genetic variants, and the harms of *not* sharing them, are numerous and well-established. Databases and mechanisms already exist to facilitate deposition and sharing of de-identified genetic variants, but clarity and transparency around best practice is needed to encourage widespread use, prevent inconsistencies between different communities, maximise individual privacy and ensure public trust. We therefore recommend that widespread sharing of a small number of genetic variants per individual, associated with limited clinical information, should become standard practice in genomic medicine. Information confirming or refuting the role of genetic variants in specific conditions is fundamental scientific knowledge from which everyone has a right to benefit, and therefore should not require consent to share. For additional case-level detail about individual patients or more extensive genomic information, which is often essential for individual clinical interpretation, it may be more appropriate to use a controlled-access model for such data sharing, with the ultimate aim of making as much information available as possible with appropriate governance.

## Recommendations

1. Open and widespread sharing of plausibly causal genetic variants with high-level disease or organ-level information via appropriate online databases should be routine clinical practice and should not be dependent upon consent from individual patients.
2. It is good practice to maintain a cryptic link to the laboratory or clinical service that shared the genetic data, so that clinical follow-up remains possible should knowledge of the implications of a variant change or to combine data to build evidence.
3. Disclosing case-level clinical detail, large variant sets or genome-wide data may be crucial for variant interpretation, accurate diagnosis or clinical management, but requires explicit consent to share openly.

## Introduction

Making an accurate diagnosis is the cornerstone of good medical practice, essential for determining prognosis, guiding treatment and informing patient management. Across all medical specialties, the interpretation of diagnostic test results relies upon knowledge of what is ‘normal’ in the population versus what ‘disease’ looks like. This knowledge relies upon sharing test results from previous patients and population controls. Without such data, the sensitivity and specificity of the test is unknown, its clinical utility is questionable, and its continued use may be harmful.

Genomic medicine is no exception to this rule, but determining what constitutes ‘normal’ and ‘disease’ can be extremely complicated and arguably the need for ongoing pooling of data is even greater than in other branches of medicine. Increasingly, clinical testing will rely on genome-wide sequencing, rather than targeted single-gene testing, and the enormous amount of normal variation in every genome ^1^ means that interpreting the results from one person’s genome requires knowledge of many thousands of other genomes across different populations and ancestral backgrounds. Despite ongoing efforts to sequence large cohorts ^2– 4^, every genome examined contains novel changes not previously seen. For diseases with a substantial genetic component, caused by a specific rare variant or variants in an individual’s genome, determining which variants are responsible for disease—and which are simply incidental, or play a minor role—is an enormous challenge. The only way to meet that challenge is by sharing data on individual variants with associated high-level disease or organ-level information that are not uniquely identifying.

## Advantages of sharing genetic variant data

**Figure 1.**
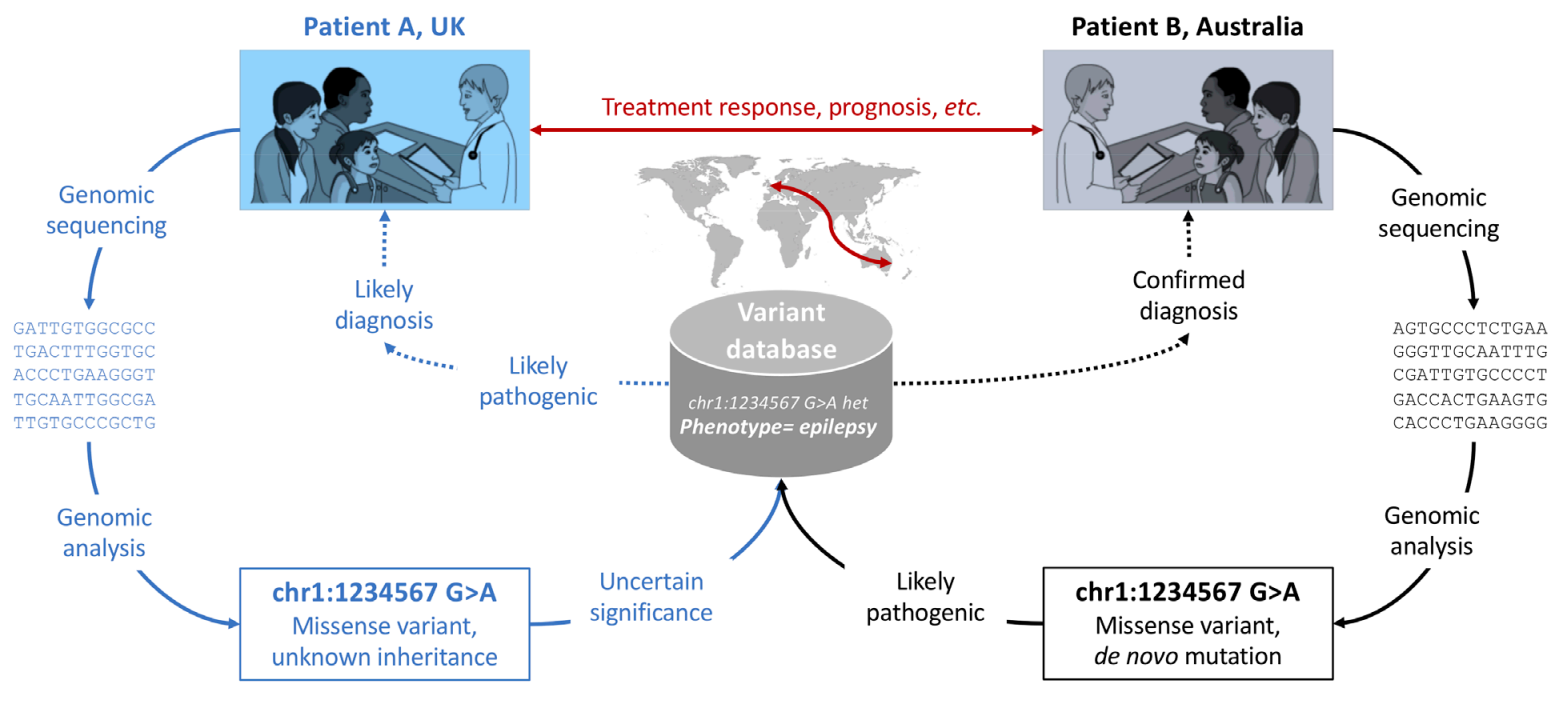
Global open variant sharing enables robust diagnoses to be made as quickly as possible; facilitating controlled sharing of detailed case-level information also informs clinical management and aids diagnosis in complex cases.

The main purpose of sharing individual genetic variants is to improve the diagnostic accuracy of genetic testing; the main data processors are clinicians and clinical scientists, and the main beneficiaries are patients and publics. Within this context, there are many benefits of sharing individual genetic variants associated with specific conditions ^5^:

1. ***Making accurate and safe diagnoses.*** Genetic testing often benefits the individual patient undergoing testing, whose diagnosis can be accurately determined and prognosis further refined. Such genetic testing is dependent on being able to compare the variant of interest to variants from thousands of other people (via a database that is accessed by the scientist or clinician doing the analysis); at a minimum, this variant comparison is necessary to characterise and usually exclude variants that are relatively common in the general population. Variants of uncertain significance are regularly generated from genome-wide testing and can most easily be resolved through being able to access and explore the context in which such variants have been observed elsewhere (see Figure 1) ^6^. Numerous examples exist where making a successful genetic diagnosis has only been possible as a result of being able to access variant and phenotype data from other individuals undergoing testing ^7– 11^, and many new genetic causes of disease have been uncovered this way ^12, 13^. While most of the published cases are clinician-led, there are an increasing number of patient-led examples of variant sharing that have also catalysed the formation of disease-specific patient support groups and created new avenues of research ^14, 15^.
2. ***More effective disease management and precision medicine.*** In some cases, an accurate genetic diagnosis leads to specific targeted therapies that can more effectively treat disease, or, in rare cases, may even reverse or prevent disease ^16– 18^. As a result of variant sharing, individuals may also be recruited to clinical trials that are tailored to their specific genotype, offering the potential for therapy where none currently exists ^19– 21^. In addition, new fundamental biological insights from genetic studies may identify novel targets for future therapies. Effective data sharing facilitates research across academia, clinical practice and industry and across different diseases and specialties ^22^.
3. ***Accurate advice for family members.*** Due to the shared familial nature of most genetic variants, the benefits of making a robust genetic diagnosis may be cascaded out to biological relatives and have a profound impact on both existing and future generations. Consideration needs to be given to if and when communication of relevant information to relatives needs to take place, and the means by which this might be facilitated ^23– 27^.
4. ***Improved understanding of genetic disease.*** There are also wider benefits to the community, including patients, clinicians and researchers across the globe, who are trying to understand and treat the causes of disease. Reporting new gene-disease associations, and sharing of variant-level information to discern which specific variants within each gene are pathogenic or benign or carry some degree of risk, is critical to advancing our understanding of genetic disease. Moreover, sharing variants together with phenotype, age and sex will allow an evolving understanding of incomplete penetrance and variable expressivity, improving interpretation of both diagnostic and predictive testing.

## Disadvantages of not sharing genetic variant data

There is a substantial opportunity cost to not sharing clinically-oriented data that could otherwise be used to accelerate medical progress. The harms of *not* sharing individual genetic variants are well established and include delayed, missed and erroneous diagnoses, leading to inappropriate care ^28– 31^ and sometimes litigation ^32, 33^. (See Box 1 and Box 2 for examples where variant sharing had a direct impact on clinical care.) Due to the familial nature of genetics, any diagnostic mistakes can easily be compounded by cascading erroneous information out to family members, thus multiplying the harms. Furthermore, without data sharing, research progress would be impeded, and the growing genomics knowledgebase—upon which the promise of personalised medicine is based—will stagnate.

Historical mistakes that exist in public variant databases ^34^ cannot be fixed without an influx of new data to allow reclassification of variants ^35, 36^, without which misdiagnoses and errors in predictive algorithms will continue. Some international databases contain wrong and erroneous variant classifications ^37^, making such curation essential. Although it has been suggested that highlighting discordance in variant interpretation can be unhelpful for clinical users ^37, 38^, exposing discordant classifications allows laboratories and clinical services to work together to understand their differences, some of which may relate to incomplete penetrance of variants, and improve concordance ^28, 39, 40^. Organisations that actively maintain private genetic variant databases, such as commercial companies that do not share variant information for proprietary reasons ^41^, are thus inhibiting diagnoses for other patients and undermining public health efforts in this area. Issues can arise where public databases are acquired by private companies, which despite being favourable for their survival may limit data access through prohibitive licencing fees.

**Box 1. *Example 1: The hazard of variant over-interpretation***

In the early 2000’s, a routine scan from a woman in her second trimester of pregnancy showed increased signal in the fetal bowel. This can be a sign of a chromosomal anomaly, viral infection or cystic fibrosis (CF) so an amniocentesis was offered. DNA analysis showed the fetus carried two *CFTR* variants that were said to be pathogenic. The parents were counselled that their baby would be affected by CF. They elected to continue the pregnancy.

After birth, the child was started on prophylactic antibiotics, twice daily physiotherapy, regular nebulisers and pancreatic supplements. Years later, the child was referred to the genetics clinic for review because the disease seemed unusually mild. **The clinical geneticist told the family that the status of one mutation had changed in the CFTR2 database and this combination was no longer thought to cause cystic fibrosis.**

As a direct consequence of this change in variant interpretation, the child’s prognosis changed from a life-limiting disorder to one of near-normal life expectancy and the day-to-day life of the child was transformed. The intensive regime of care was substantially reduced.

**Box 2. *Example 2: The need for population-specific variation data***

A middle-aged Turkish man was referred to clinical genetics because he had colorectal cancer and numerous polyps were discovered at surgery. A homozygous variant in *MUTYH* was identified and reported to be of “unknown significance” in the diagnostic laboratory report. Biallelic *MUTYH* mutations cause MUTYH-associated polyposis (MAP), a recessive syndrome consistent with the diagnosis. Specific mutations are found at different frequencies in different populations.

Evaluation of available databases revealed that the variant had been identified once before in a patient with colon cancer and polyposis. Notably this second patient was also Turkish. No functional data were available and *in silico* analyses were inconclusive. The variant is extremely rare; present in only 7 individuals, all of South or East Asian origin, in the Exome Aggregation Data set of 61,486 individuals. However, no Turkish samples are listed as contributing to any of these datasets and no *MUTYH* or exome data from the general Turkish population is available.

Thus it is unclear whether this *MUTYH* variant is a pathogenic Turkish founder mutation or a non-pathogenic variant that is particularly prevalent in the Turkish population, but rare/absent in other populations. This lack of clarity presents significant clinical challenges in managing the patient and his relatives. **Sharing data generated in laboratories worldwide and across more ethnic groups would provide information to differentiate between these options and would allow clear classification of this and many other variants and reduce the potential for health disparities.**

## Perceived harms of sharing genetic variant data

We have not been able to find any evidence that sharing data relating to individual genetic variants in the context of clinical applications causes harm. Nonetheless, perceived harms include re-identification of individuals across different datasets, loss of security of associated medical information (about the individual or their relatives), and the maleficent misuse of data ^42, 43^. Early fears relating to genetic discrimination and the impact of genetic data on insurance premiums have not materialised in the UK and many other countries, thanks in part to genetic non-discrimination legislation and the Code on Genetic Testing and Insurance ^44, 45^. Identification of an individual through knowledge of their genetic variant(s) is now perhaps the main concern. Although it is never possible to guarantee anonymity, and no data sharing system can be 100% secure, individual genetic variants—even very rare ones—are not uniquely identifying, and re-identification would require an intimate knowledge of the individual’s genotype or phenotype together with some information to trace that genotype/phenotype to a specific person. In practice, only an individual patient or their clinician would easily be able to re-identify themselves from a specific variant, neither of which would constitute a breach of confidentiality ^46^. A related concern is the perception that all genetic data are personal and therefore inherently sensitive, which stems from conflating genome-wide data with individual genetic variants.

## Finding a balance

In our view, the definite and provable harms of *not* sharing genetic data outweigh the potential and largely hypothetical harms of sharing, a view that is corroborated by several recent litigation cases ^32, 33^ and supported by several large opinion surveys ^47, 48^. Some empirical research has shown that patients and research participants support widespread data sharing ^48, 49^ and believe that the positive consequences outweigh the potential negatives ^47^. Clinical experience also suggests that, when the risks and benefits are explained to them and when invited to give consent, most patients are keen for their variant data and associated phenotypes to be shared. Recognising these benefits, 13 European countries have recently signed a declaration for delivering cross-border access to their genomic information. Nonetheless, in our increasingly data-aware society, there is a perception that data sharing is inherently risky ^50^. A balance must therefore be struck between sharing sufficient data to reap the benefits, but only as much data as is needed to avoid the potential (perceived and actual) harms.

We have previously proposed a principle of proportionality in genetic data sharing, that balances the depth of data shared with the breadth of sharing ^51^. With any dataset, decisions must be made about what specifically to share and how widely to share it. Many of the clinical benefits of data sharing in genetics can be realised by sharing a tiny subset of an individual’s de-identified genetic variants ^52^, together with limited medical data, rather than necessarily whole genomes. This principle is in accordance with data privacy laws such as the new European General Data Protection Regulation (GDPR), which mandates that stored data are “ *adequate, relevant and limited to what is necessary in relation to the purposes for which they are processed*” ^53^.

The specifics of implementation are critical and agreeing standards for sharing variants and associated clinical data is essential. Specific data elements for sharing individual genetic variants have been outlined previously ^54^ and include (see Table 1):

1. a standardised genetic description of the variant(s), including Human Genome Variation Society (HGVS) nomenclature and genomic coordinates of the variant;
2. the variant classification and summary of evidence upon which that assertion was based;
3. the disease and inheritance pattern (e.g. dominant/recessive) upon which the clinical significance was asserted;
4. a standardised clinical description of the high-level disease phenotypes in the patient(s) that are included as supporting observations for the variant assertion, using appropriately controlled vocabulary/ontology; and
5. a cryptic or hidden link to the laboratory or clinical service that submitted the data, to enable further information to be requested and avoid data duplication but obscure the precise geographical location.

**Table 1.** Example of genomic variant sharing.

	Variant 1	Variant 2
**Variant**	Standardised description of variant, including genomic coordinates	Standardised description of variant, including genomic coordinates
**Gene**	*e.g. MYH7*	*e.g. MYH7*
**Genotype**	Heterozygous	Heterozygous
**Phenotype**	Hypertrophic cardiomyopathy	Hypertrophic cardiomyopathy
**ACMG/AMP variant-level** **evidence ^55^**	PS1 – a different variant at the same position has previously been established to be pathogenic PM1 – occurs in the head of the protein (a functional domain with high probability pathogenicity) PM2 – absent from the general population PP3 – computational evidence suggests deleterious effect on gene product	PM1 – occurs in the head of the protein (a functional domain with high probability pathogenicity) PM2 – absent from the general population PP3 – computational evidence suggests deleterious effect on gene product
**Interpretation (based on** **public data)**	Likely pathogenic	Variant of uncertain significance
**Aggregated case-level** **evidence**	Observed in 1/10,000 individuals referred with diagnosis of HCM	Lab A – variant observed in 2/3,000 total cardiomyopathy patients sequenced Lab B – 2/4,000 Lab C – 1/3,000 Lab D – 1/1,000 patients
**Interpretation (with** **variant sharing)**	Likely pathogenic	Likely pathogenic

We recommend that openly sharing variant-level data, such as that included in Table 1, should be routine practice. No personal identifiers should be openly shared (e.g. name, hospital IDs, address, etc), and only the minimal genetic and clinical information required (as outlined in the five points above) to assist with interpreting a similar variant should be included. We recommend a cryptic link to the individual case-level data is maintained in a de-identified fashion via the laboratory or clinical service that submitted the data, that may obscure its precise geographical origin by deposition via another platform, to enable clinical follow-up if needed. Linking basic clinical information with information about genetic variation is crucial for supporting variant interpretation and aiding diagnoses. However, as with more extensive genome-wide data, or genomic risk scores, different levels of clinical detail will require different modes of sharing, i.e. open versus controlled access. Controlled sharing of more detailed phenotypes allows for more accurate diagnosis by enabling an independent evaluation of the clinical fit; if a diagnosis is simply stated in association with a variant, the validity of that association cannot be evaluated. Including this detailed clinical information with a genetic test result also avoids potential attrition, where individual clinicians need to go back to the original data generator to obtain sufficient information with which to make a diagnosis in their patient.

A flexible platform with broad international sharing of variant data together with national/local sharing of more granular phenotypic data would enable both needs to be addressed. Numerous databases already exist for collating and sharing genetic information, which may have differing requirements for data deposition and thus offer different advantages and disadvantages. For example, US-based ClinVar ^56, 57^ is one of the largest genetic variant deposition databases, with >600,000 open access variants assayed primarily through laboratory genetic testing services, of which 60% of the >170,000 pathogenic/likely pathogenic variants have at least some supporting evidence, either as a written evidence summary and/or PubMed citations. UK-based DECIPHER ^10, 58, 59^ is a global platform containing detailed case-level clinical data associated with >65,000 variants, of which 90% of pathogenic/likely pathogenic variants have associated phenotypes. DECIPHER uses a tiered access model whereby around half the cases are open access and half are accessible to members of closed groups to enable data-sharing that is compliant with local or national governance requirements. DECIPHER and many other variant databases internationally are now part of Matchmaker Exchange (MME), which was created to address the issue of data siloes by establishing *“a federated network connecting databases of genomic and phenotypic data using a common application programming interface*” ^7, 8^. MME has facilitated gene discoveries that would not have been possible were the data from individual rare disease patients siloed in individual databases (see https://www.matchmakerexchange.org/statistics.html).

## Establishing good practice

Uncertainty about what are permissible types of genetic variant sharing and when explicit consent is required means that current data sharing practices across regional genetics centres are highly variable ^46^. The inclusion of genetic data within Article 9 of the European GDPR, “ *Processing of special categories of personal data*”, has created further confusion about the legality of sharing individual variants. There is therefore a need to establish and agree best practice ^60^ for data sharing within genomic medicine, to avoid inconsistent practices across different regions, communities and jurisdictions, and ensure transparency and consistency when speaking to patients. Genetic variant data of the sort described above does not meet a recently proposed Data Sharing Privacy Test ^61^, as the data is neither inherently sensitive nor uniquely identifying. Within the UK, the National Data Guardian has stated that “ *the duty to share information can be as important as the duty to protect patient confidentiality*” ^62^, a principle that applies to all data generated across the UK National Health Service. The American College of Medical Genetics and Genomics recently published a position statement in 2017 that “ *laboratory and clinical genomic data sharing is crucial to improving genetic health care*” ^63^. However, genomic medicine is inherently a global enterprise, so more countries need to follow suit ^64^. The approach to data sharing espoused by the Global Alliance for Genomics and Health ^65, 66^ is rooted in international human rights legislation, focussing on our ‘solidarity rights’ to genomic information ^67, 68^ and emphasising the social good that can derive from appropriate data sharing. The handful of patients with the same rare diagnosis may be scattered across different countries, and are therefore best served when data are shared as openly and as widely as possible. Patients across the globe currently benefit from shared data and derived knowledge in databases such as ClinVar, DECIPHER and the Leiden Open Variation Database (LOVD) ^69^. Services that are not currently sharing their clinical data owe a substantial data debt and risk perpetuating current data biases.

## Explicit consent should not be required for individual variant sharing

A recent analysis of the ethical principles that should guide genomic medicine services suggested that the “ *use of genomic data for the advancement of medical knowledge should be permitted without explicit consent*" ^70^. In addition to variants from current and future patients, in whom the benefits of sharing vastly outweigh the potential harms, enormous swathes of legacy data exist from decades of patients who have undergone genetic testing. Some of these individuals are no longer alive and most are no longer in touch with their clinicians, making obtaining consent for data sharing impossible. Sharing variants from these tests could potentially benefit many thousands of patients without posing any risk of harm to the data subjects.

Although considering ownership of data has often been used as a route to determine what can be done with it, examining who controls access to the data is perhaps a more useful way forward than entering into ownership debates which, even if resolved, would not answer the question of what can legitimately be done with the data ^71^. Individuals have a right to control access to data relating to them, but when it is not uniquely identifying and can benefit others without harming the individual—as is the case for genetic variants—rights of veto should be limited to the most unusual situations. A link between a particular genetic variant and associated disease is not personal information any more than the link between high blood cholesterol and heart disease, for example.

We therefore propose that patient consent should *not* be required in order to share variant-level data on individual genetic variants, with minimal disease information ^54^. Agreeing this principle of “clinical variant-level sharing” ^54^ would remove the onus from data generators to ensure that they have the appropriate consents and permissions in place, and replace it with an unambiguous policy that is clear and transparent for both data generators and data subjects. In addition, we suggest that more detailed case-specific information generated within a particular healthcare system should initially remain within that healthcare system, sensitive to the quirks of each individual regulatory regime, but with the aim of eventual open data sharing following discussion with the patient and subject to their explicit consent.

## Conclusions

All interpretation of genetic data is fundamentally dependent upon data sharing, since it is rarely possible to robustly demonstrate an association between a particular genetic change and a disease with an “N-of-one”. Therefore, sharing genetic variant data—albeit aggregated at some level and de-identified as far as possible—is inseparable from the practice of genomic medicine. Clinicians cannot treat patients appropriately if they cannot compare their patient’s data with data from healthy populations and other patients to establish a safe genetic diagnosis. It is therefore beholden upon those who generate and interpret genetic test results to allow access to relevant data as widely and as openly as possible, by depositing the data into appropriate databases and making it available to others to access whilst remaining compliant with local and national legislation and data governance. Numerous databases exist with aggregated genetic information, and although they differ in their deposition requirements and governance structures, ensuring interoperability between them through initiatives such as Matchmaker Exchange will prevent information silos and ensure longer-term sustainability.

Despite the overwhelming benefits of genetic variant sharing, and paucity of proven harms, there remain anxieties around deposition of individual genetic variants to open access databases. We propose that consent should not be required for widespread, open sharing of individual de-identified genetic variants linked with high-level phenotypes (i.e. associated disease or organ-level information), and that sharing such data should become standard practice in genomic medicine. We also recommend that richer case-level phenotypic detail (such as individual phenotype terms with age and other case-specific information) is shared within healthcare systems to facilitate robust diagnosis and that consent is routinely sought at the time of diagnosis to share such data openly. Ultimately, both the promise and the safety of genomic medicine will depend on our ability and willingness to share.

## Data availability

No data are associated with this article

## Acknowledgments

The authors wish to thank Fiona Cunningham, Ewan Birney, Matthew Hurles, David FitzPatrick, Graeme Black and Patrick Chinnery for helpful comments and input on this manuscript.
