# Supplementary material for: Genomic variant sharing: a position statement
Source: Wellcome Open Res. 2019 Dec 4;4:22. Originally published 2019 Feb 5. [Version 2] doi: 10.12688/wellcomeopenres.15090.2 (PMC6913213; doi:10.12688/wellcomeopenres.15090.2)
Supplement: Peer review history

# Peer review: Reviewer response for version 2

**DOI:** 10.21956/wellcomeopenres.17121.r37289

**Published:** 2019-12-12

**Authors:** Gert Matthijs

**Affiliations:** 1 Center for Human Genetics, KU Leuven, Leuven, Belgium

I thank the authors for constructively replying to my comments by clarifying all issues and complementing the manuscript where necessary. I have no further comments. As a community, we are indebted to the authors for sharing views and publishing a position statement on variant sharing.

I confirm that I have read this submission and believe that I have an appropriate level of expertise to confirm that it is of an acceptable scientific standard.

# Peer review: Reviewer response for version 1

**DOI:** 10.21956/wellcomeopenres.16463.r34792

**Published:** 2019-03-11

**Authors:** Christa L Martin, Erin Rooney Riggs, Heidi Rehm

**Affiliations:** 1 Autism & Developmental Medicine Institute (ADMI), Geisinger Health System, Danville, PA, USA; 2 Geisinger Medical Center, Danville, PA, USA; 3 Massachusetts General Hospital, Boston, MA, USA; 4 Broad Institute of MIT and Harvard, Cambridge, MA, USA; 5 Brigham & Women’s and Harvard Medical School, Boston, MA, USA

**Answers to the questions:**

 1. Is the rationale for the Open Letter provided in sufficient detail?

 The authors state that the rationale for their recommendation to share genomic variants with limited clinical information is to encourage consistency and transparency among the genetics community, and to bolster the practice of genomic medicine, making it more beneficial to patients.

 2. Does the article adequately reference differing views and opinions?

 The article does provide a section outlining “perceived harms of sharing genetic variant data,” effectively outlining commonly proposed concerns about genomic data sharing. To be clear, our group, the Clinical Genome Resource (ClinGen), has also published articles with similar recommendations to those put forth by these authors on genomic data sharing, and we agree with the concepts presented in this manuscript. However, we are aware of at least one other “differing” opinion that was not represented here: the opinion that public data sharing highlights discordance in variant interpretation and is potentially confusing for clinical users ^1^^,^^2^. Our group believes that exposing discordant classifications between laboratories is actually a *benefit* to data sharing, allowing laboratories to see where they differ and work together towards concordance ^3^^,^^4^^,^^5^.

 3. Are all factual statements correct, and are statements and arguments made adequately supported by citations?

 In our general response, we note one place where we thought a factual statement was not completely accurate. The authors state: “....US-based ClinVar is perhaps the leading genetic variant deposition database.. but most variants have only very limited or no clinical information and no supporting evidence associated with them.” While it is true that most entries do not contain patient data, the majority (62%) of the more than 170,000 pathogenic/likely pathogenic variants in ClinVar have supporting evidence, either as written evidence summaries and/or PubMed citations.

 There were also some other places in the document where additional context would be useful (for example, in order for the reader to understand the nuances between variant-level and case-level data sharing or to further explain the difference between the disease upon which a claim of variant pathogenicity was made and the phenotypic features presenting in an individual patient). These issues are noted in our general response.

 4. Is the Open Letter written in accessible language?

 Yes

 5. Where applicable, are recommendations and next steps explained clearly for others to follow?

 Providing more clarity would be helpful regarding “next steps” for readers to follow, particularly in regards to where variant information could be submitted.

**General report:**

 Wright and colleagues have written an open letter to address genomic variant sharing. It is a thorough and excellent accounting of the rationale for this type of data sharing and we commend the authors for taking the time to thoughtfully review and provide guidance on this important topic. We have a few suggestions and some minor edits that could strengthen the article and provide additional guidance to the community.

**Higher level comments and suggestions:**

 In the first section under “Recommendations” the first recommendation suggests only sharing “plausibly causal” genetic variants. We think this recommendation is insufficient and strongly encourage this guidance to include sharing of *all* variants that have been reviewed. The literature and databases are currently littered with false claims of causality/pathogenicity. It is critically important that we also share evidence on variants that are deemed benign or uncertain, or, at the case-level, deemed non-causal. Over three-quarters of ClinVar’s content is made up of variants classified as benign, likely benign, or variant of uncertain significance (VUS) and this data has been enormously useful to counter many of the false claims of pathogenicity from the literature.

 In addition, there is a bit of conflating of the concept of variant-level versus case-level interpretation and it would be useful to better separate these concepts in the paper. We have previously defined “variant-level” information as the aggregation of all evidence and observations to define the pathogenicity of a variant (i.e., its capacity to cause disease) ^6^. This may include evidence from a current case under investigation, but also takes into account all prior available data. However, whether a given variant is actually casual for the symptoms in a given patient is best called case-level interpretation and involves additional factors such as penetrance, a phenotype match with the relevant gene, and allelic information (e.g., recessive disease requires two alleles).

 Related to this issue, we recommend in the section that outlines five specific data elements and references our prior publication ^6^, that items 2 and 4 be swapped to start with the variant level claim and then include the patient phenotype as part of the supporting evidence. This approach is more in line with variant-level data sharing and our referenced publication, which should be distinguished from case-level sharing, which is also important, but requires additional considerations as the authors have pointed out. Similarly, the variant claim (e.g., pathogenic, benign) should be asserted against a disease, not the patient’s clinical features, which should be left to the case-level interpretation step. We have made suggested edits below to data elements 3 and 4 to better clarify these points (additions in bold, deletions indicate by strikethrough):

*“Specific data elements for sharing individual genetic variants have been outlined previously ^42^ and include (see Table 1):*

*1. a standardised genetic description of the variant(s), including Human Genome Variation Society (HGVS) nomenclature and genomic coordinates of the variant;*

*2. the clinical significance and summary of evidence upon which that assertion was based;*

*3. the **disease and** inheritance pattern of the disease (e.g. dominant/recessive) **upon which the clinical significance is asserted**;*

*4. a standardised clinical description **of any of **the clinical features in the patient **(s)that are included as supporting observations for the variant assertion**, using appropriately controlled vocabulary/ ontology;and*

*5. a cryptic link to the laboratory or clinical service that submitted the data (to enable further information to be requested and avoid data duplication).”*

 Next, the authors state, “We recommend a cryptic link to the individual case-level data is maintained in a de-identified fashion via the laboratory or clinical service that submitted the data, that may obscure its geographical location by deposition via another platform, to enable clinical follow-up if needed.” We think this topic requires further consideration of the benefits and drawbacks of obscuring the submitter’s location. For most laboratories that perform a large volume of testing and receive samples from geographically diverse locations, it seems unnecessary to obscure the geographical location of the laboratory and data; indeed, the geographical location of the laboratory is easily discernible given that individual variants are attributed to specific laboratories in databases, such as ClinVar. ClinVar has operated with transparency to the submitter and their location without harm for several years now. To the contrary, it can be helpful to recognize the potential for data duplication, which is not uncommon. We would suggest a more nuanced discussion of this topic. For example, one may consider obscuring geographical location only in instances where the population is small or geographically isolated.

 Finally, it would be useful if the authors gave more concrete suggestions for where laboratories should submit their classified variants today. Do the authors support that direct submission to ClinVar is one recommended option? The authors describe both ClinVar and DECIPHER, but it is unclear what their recommendation would be. Given the momentum that ClinVar has achieved, it seems important that wherever the variant classifications are initially generated and stored, that they also be easily submitted to ClinVar. DECIPHER does have the advantage of a richer connection to case-level data. If DECIPHER took on a role as an additional site of primary variant deposition (not clear if it accepts individual submitted variant interpretations), we would assume the authors would agree that it would still be important for DECIPHER to facilitate submission to ClinVar on behalf of its users, in the same way that DECIPHER is able to fully consume ClinVar data. Would this be a second recommended option? More detail around any recommendations and/or future plans would likely be useful to readers.

**Minor suggestions and edits:**

 In the abstract the authors state: “We therefore recommend that widespread sharing of a small number of individual genetic variants associated with limited clinical information should become standard practice in genomic medicine.” We assume the authors mean a small number “per individual” but we read it as stating that each “source/laboratory” should only share a small amount of data, in general. We suggest deleting “a small number of” and saving the nuance of per individual issue for later in the paper. Alternatively, you could reword the statement to read: “We therefore recommend that widespread sharing of a small number of an individual’s genetic variants associated with limited clinical information should become standard practice in genomic medicine.” This same issue occurs in the second paragraph of the section “Finding a balance”. In the sentence “...sharing a tiny subset of..” we suggest adding “an individual’s” after “of”.

 In the last sentence of the abstract the authors state “For additional case-level detail about individual patients or more extensive genomic information, which is often essential for clinical interpretation, it may be more appropriate to use a controlled-access model for data sharing…..”. We fear this could be implied as abandoning the core suggestion if one wants to also share case-level data and therefore we suggest clarifying by adding “this additional” so the sentence reads “...it may be more appropriate to use a controlled-access model for this additional data sharing…..”

 For the second Recommendation “A single genetic variant is not personally identifiable

 information; however, it is good practice to maintain a cryptic link to the laboratory or clinical service that shared the genetic data so that clinical follow-up remains possible should knowledge of the implications of a variant change.” We suggest adding “or to combine data to build evidence”. In our experience, many variants change classifications once labs bring their evidence/observations together, but a source for contact is needed to communicate and bring the data together.

 Another good example of patient benefit, and avoidance of harm, from data sharing is Grant et al, referenced below, in case you would like to cite ^7^.

 The authors state: “....US-based ClinVar is perhaps the leading genetic variant deposition database…….but most variants have only very limited or no clinical information and no supporting evidence associated with them.” This statement is not completely accurate. While it is true that most entries do not contain patient data, the majority (62%) of the >170,000 pathogenic/likely pathogenic variants have supporting evidence, either as a written evidence summary and/or PubMed citations.

We confirm that we have read this submission and believe that we have an appropriate level of expertise to confirm that it is of an acceptable scientific standard.

## References

[1] : Clinical Variant Classification: A Comparison of Public Databases and a Commercial Testing Laboratory. *Oncologist*.22(7) : 10.1634/theoncologist.2016-0431 797-803 10.1634/theoncologist.2016-0431 28408614PMC5507641

[2] : Comparison of locus-specific databases for BRCA1 and BRCA2 variants reveals disparity in variant classification within and among databases. *J Community Genet*.2015;6(4) : 10.1007/s12687-015-0220-x 351-9 10.1007/s12687-015-0220-x 25782689PMC4567983

[3] : Scaling resolution of variant classification differences in ClinVar between 41 clinical laboratories through an outlier approach. *Hum Mutat*.2018;39(11) : 10.1002/humu.23643 1641-1649 10.1002/humu.23643 30311378PMC6188667

[4] : Clinical laboratories collaborate to resolve differences in variant interpretations submitted to ClinVar. *Genet Med*.19(10) : 10.1038/gim.2017.14 1096-1104 10.1038/gim.2017.14 28301460PMC5600649

[5] : Copy number variant discrepancy resolution using the ClinGen dosage sensitivity map results in updated clinical interpretations in ClinVar. *Hum Mutat*.2018;39(11) : 10.1002/humu.23610 1650-1659 10.1002/humu.23610 30095202PMC7374944

[6] : Points to consider for sharing variant-level information from clinical genetic testing with ClinVar. *Cold Spring Harb Mol Case Stud*.4(1) : 10.1101/mcs.a002345 10.1101/mcs.a002345 29437798PMC5793773

[7] : Rapid communication of efforts to resolve differences or update variant interpretations in ClinVar through case-level data sharing. *Cold Spring Harb Mol Case Stud*.2018;4(5) : 10.1101/mcs.a003467 10.1101/mcs.a003467 30275005PMC6169822

# Response

**Published:** 2019-12-02

**Authors:** Caroline Wright

**Affiliations:** University of Exeter, UK

Wright and colleagues have written an open letter to address genomic variant sharing. It is a thorough and excellent accounting of the rationale for this type of data sharing and we commend the authors for taking the time to thoughtfully review and provide guidance on this important topic.

***We thank the reviewer for their positive comments.***

 We are aware of at least one other “differing” opinion that was not represented here: the opinion that public data sharing highlights discordance in variant interpretation and is potentially confusing for clinical users ^1^ ^,^ ^2^. Our group believes that exposing discordant classifications between laboratories is actually a *benefit* to data sharing, allowing laboratories to see where they differ and work together towards concordance ^3^ ^,^ ^4^ ^,^ ^5^.

***This is a good point and we agree with the reviewer. We have added a sentence about this into the manuscript with these additional references.***

 In the first section under “Recommendations” the first recommendation suggests only sharing “plausibly causal” genetic variants. We think this recommendation is insufficient and strongly encourage this guidance to include sharing of *all* variants that have been reviewed. The literature and databases are currently littered with false claims of causality/pathogenicity. It is critically important that we also share evidence on variants that are deemed benign or uncertain, or, at the case-level, deemed non-causal. Over three-quarters of ClinVar’s content is made up of variants classified as benign, likely benign, or variant of uncertain significance (VUS) and this data has been enormously useful to counter many of the false claims of pathogenicity from the literature.

***See earlier comment about this and our concerns around linking a variant to a phenotype that it does not cause.***

 In addition, there is a bit of conflating of the concept of variant-level versus case-level interpretation and it would be useful to better separate these concepts in the paper. We have previously defined “variant-level” information as the aggregation of all evidence and observations to define the pathogenicity of a variant (i.e., its capacity to cause disease) ^6^. This may include evidence from a current case under investigation, but also takes into account all prior available data. However, whether a given variant is actually casual for the symptoms in a given patient is best called case-level interpretation and involves additional factors such as penetrance, a phenotype match with the relevant gene, and allelic information (e.g., recessive disease requires two alleles).

***We have tried to clarify this throughout the text.***

 Related to this issue, we recommend in the section that outlines five specific data elements and references our prior publication ^6^, that items 2 and 4 be swapped to start with the variant level claim and then include the patient phenotype as part of the supporting evidence. This approach is more in line with variant-level data sharing and our referenced publication, which should be distinguished from case-level sharing, which is also important, but requires additional considerations as the authors have pointed out. Similarly, the variant claim (e.g., pathogenic, benign) should be asserted against a disease, not the patient’s clinical features, which should be left to the case-level interpretation step. We have made suggested edits below to data elements 3 and 4 to better clarify these points (additions in bold, deletions indicate by strikethrough):

*“Specific data elements for sharing individual genetic variants have been outlined previously ^42^ and include (see Table 1):*

*1. a standardised genetic description of the variant(s), including Human Genome Variation Society (HGVS) nomenclature and genomic coordinates of the variant;*

*2. the clinical significance and summary of evidence upon which that assertion was based;*

*3. the **disease and** inheritance pattern of the disease (e.g. dominant/recessive) **upon which the clinical significance is asserted**;*

*4. a standardised clinical description **of any of **the clinical features in the patient **(s)that are included as supporting observations for the variant assertion**, using appropriately controlled vocabulary/ ontology;and*

*5. a cryptic link to the laboratory or clinical service that submitted the data (to enable further information to be requested and avoid data duplication).”*

***We have made these changes.***

 Next, the authors state, “We recommend a cryptic link to the individual case-level data is maintained in a de-identified fashion via the laboratory or clinical service that submitted the data, that may obscure its geographical location by deposition via another platform, to enable clinical follow-up if needed.” We think this topic requires further consideration of the benefits and drawbacks of obscuring the submitter’s location. For most laboratories that perform a large volume of testing and receive samples from geographically diverse locations, it seems unnecessary to obscure the geographical location of the laboratory and data; indeed, the geographical location of the laboratory is easily discernible given that individual variants are attributed to specific laboratories in databases, such as ClinVar. ClinVar has operated with transparency to the submitter and their location without harm for several years now. To the contrary, it can be helpful to recognize the potential for data duplication, which is not uncommon. We would suggest a more nuanced discussion of this topic. For example, one may consider obscuring geographical location only in instances where the population is small or geographically isolated.

***This is an interesting point, but a more nuanced discussion is outside the scope of this paper. We have changed the text to suggest that the “precise” geographic location should be obscured, but have not discussed it in further detail as this issue will vary between countries depending upon the catchment area of the testing laboratory.***

 Finally, it would be useful if the authors gave more concrete suggestions for where laboratories should submit their classified variants today. Do the authors support that direct submission to ClinVar is one recommended option? The authors describe both ClinVar and DECIPHER, but it is unclear what their recommendation would be. Given the momentum that ClinVar has achieved, it seems important that wherever the variant classifications are initially generated and stored, that they also be easily submitted to ClinVar. DECIPHER does have the advantage of a richer connection to case-level data. If DECIPHER took on a role as an additional site of primary variant deposition (not clear if it accepts individual submitted variant interpretations), we would assume the authors would agree that it would still be important for DECIPHER to facilitate submission to ClinVar on behalf of its users, in the same way that DECIPHER is able to fully consume ClinVar data. Would this be a second recommended option? More detail around any recommendations and/or future plans would likely be useful to readers.

***We do not wish to prescribe where users should deposit their data, as many databases offer slightly different features. Instead, we support the federation of databases using systems such as MME, to ensure that data are not siloed. We have added a sentence about MME.***

**Minor suggestions and edits:**

 In the abstract the authors state: “We therefore recommend that widespread sharing of a small number of individual genetic variants associated with limited clinical information should become standard practice in genomic medicine.” We assume the authors mean a small number “per individual” but we read it as stating that each “source/laboratory” should only share a small amount of data, in general. We suggest deleting “a small number of” and saving the nuance of per individual issue for later in the paper. Alternatively, you could reword the statement to read: “We therefore recommend that widespread sharing of a small number of an individual’s genetic variants associated with limited clinical information should become standard practice in genomic medicine.” This same issue occurs in the second paragraph of the section “Finding a balance”. In the sentence “...sharing a tiny subset of..” we suggest adding “an individual’s” after “of”.

***We have made these changes.***

 In the last sentence of the abstract the authors state “For additional case-level detail about individual patients or more extensive genomic information, which is often essential for clinical interpretation, it may be more appropriate to use a controlled-access model for data sharing…..”. We fear this could be implied as abandoning the core suggestion if one wants to also share case-level data and therefore we suggest clarifying by adding “this additional” so the sentence reads “...it may be more appropriate to use a controlled-access model for this additional data sharing…..”

***We have made this change.***

 For the second Recommendation “A single genetic variant is not personally identifiable

 information; however, it is good practice to maintain a cryptic link to the laboratory or clinical service that shared the genetic data so that clinical follow-up remains possible should knowledge of the implications of a variant change.” We suggest adding “or to combine data to build evidence”. In our experience, many variants change classifications once labs bring their evidence/observations together, but a source for contact is needed to communicate and bring the data together.

***We have made this change.***

 Another good example of patient benefit, and avoidance of harm, from data sharing is Grant et al, referenced below, in case you would like to cite ^7^.

***We have added this reference.***

 The authors state: “....US-based ClinVar is perhaps the leading genetic variant deposition database…….but most variants have only very limited or no clinical information and no supporting evidence associated with them.” This statement is not completely accurate. While it is true that most entries do not contain patient data, the majority (62%) of the >170,000 pathogenic/likely pathogenic variants have supporting evidence, either as a written evidence summary and/or PubMed citations.

***Thank you, we have updated the text with this information.***

# Peer review: Reviewer response for version 1

**DOI:** 10.21956/wellcomeopenres.16463.r34794

**Published:** 2019-03-08

**Authors:** Gert Matthijs

**Affiliations:** 1 Center for Human Genetics, KU Leuven, Leuven, Belgium

Correct genomic variant interpretation and classification are an important and often complex issue, not only in research, but also, and even more critically, in genetic diagnostics and clinical care. Variant interpretation relies on different criteria and different in silico tools are available.

 The authors rightly argue that sharing of genomic variants is essential for improving and facilitating variant interpretation. Indeed, patients will strongly benefit from open and well-managed databases. Databases may be federated, as long as a swift and controlled exchange of information is available.

 Clearly, variant sharing is more than a clinical or technical issue, it is a societal issue: citizen – and not just patients – should be informed about the necessity and value of variant sharing. They should be convinced that variant sharing is essential and safe. It is a matter of solidarity and mutual interest to share data as broadly as possible. The principle of proportionality, in relation to potential harm, is certainly rightly applicable to variant sharing.

 Comment on the abstract and beyond:

- The authors use ‘de-identified’ and ‘pseudonomised’, ‘cryptic link’ and a few other descriptions. It would be good to select the best term or definition and explain it to the readership.
- It is unclear what is meant in the abstract with “a small number of …”. Small is hard to define.
- “Information robustly linking genetic variants with specific conditions is fundamental biological knowledge.” This is a significant statement that should be explored and explained in more detail, especially if the statement adds that it “should not require consent…”.

 

 On the recommendations:

- In recommendation 1. The ‘small number’ from the abstract is not well reflected (or vice versa). What are ‘high level’ phenotypes, and why would sharing be limited to these?
- Recommendation states no consent in 1. and explicit consent in 3. This dichotomy is not presented in the Abstract. Again, the definition of ‘small’ is crucial, in all instances of policy, defining a ‘cut-off’ is a tricky thing.
- In general, what about sharing genomic variant that are excluded from disease, i.e. definitely not linked to the disease? The best example is in trans with a known dominant, pathogenic mutation. That information is equally useful.

 

 On the Advantages:

 For 2. Clearly, individuals may be identified in data bases by the genotype, for inclusion in clinical trials. With whom shall the data be shared? Companies? What would be the conditions? Who shall be the custodian? How to warrant and permit access? It would be nice to elaborate a bit on this. It is another aspect of variant sharing, that is not covered under the umbrella of variant interpretation.

 For 3. The moral duty to help has been turned into a legal obligation in France. It may be interesting to cite this, as it is an example or situation that may pop up in other countries.

 For documentation of the situation in France, please visit the following sites:

https://www.legifrance.gouv.fr/affichCodeArticle.do?cidTexte=LEGITEXT000006072665&idArticle=LEGIARTI000027594214&dateTexte=&categorieLien=cid https://www.legifrance.gouv.fr/affichTexte.do?cidTexte=JORFTEXT000027592003&categorieLien=id

https://www.legifrance.gouv.fr/affichTexte.do?cidTexte=JORFTEXT000029921462

https://www.legifrance.gouv.fr/affichTexte.do?cidTexte=JORFTEXT000027592025&categorieLien=id

 For 4. How shall data be linked to natural history of disease, or vice versa?

 On the Disadvantages:

- Ref 29 is not tightly linked to the issue of the proposed international sharing of data. Are there other cases/references?
- There are other, early papers on re-classification, e.g. Piton et al. 2013 ^1^.
- It is probably worthwhile to mention that some international databases are ‘contaminated’ i.e. contain a wrong and erroneous variant classification. So the curation is essential. Variant database should explicit how the data is collected and managed. In the diagnostic arena, there is a consensus that HGMD data should be explicitly double-checked.
- The authors also mention the issue of private databases. It is unfortunate indeed that genetic and genomic analyses that are performed in often commercial laboratories do not make it to the public databases. Several large laboratories, mostly in the US, are committed to sharing data, like for instance via ClinVar. However, bad examples do exist as well, and have been denounced early on.
  References could be added, e.g. Conley et al 2014 ^2^.
  Also of note is that several databases, that were originally open, have been acquired by commercial companies. The latter has been favourable for their survival, however, the licencing fees are often prohibiting individual laboratories to obtain access. Equally, some companies offer access to their own clinical diagnostic databases, but again, the prices are mostly prohibitive. The data in private databases, especially these that are well curated, may be considered as having a value – as a result of intellectual or other efforts to generate good data – and thus come with a price. How to deal with this?
- In parallel, the public laboratories have not been very active in submitting variants. What kind of incentive would be needed to promote data sharing?
- The statement on informed consent hints at an important shift in the policy of Decipher to request consent. This policy was reportedly very strict in the early days. The position statement pleas for a relaxed (or no) requirement for a written consent. It would be interesting to read how and why the policy has evolved so significantly.

 

 Finding a balance:

- What is the link between the text and Table 1. Table 1 does not list all the elements that are listed in the text. It would be good to explain to the reader what the aim of Table 1 is.
- Open versus controlled access: open access shall best be promoted, given the large number of labs that will either submit or consult.
- Open access databases are not necessarily free. Are there any other incentives to urge (diagnostic or research) laboratories to share variants? The latter are invited to submit in relation to publication, the former? Linking it to reimbursement of the test would be an option for laboratories operating in a ‘fee for service’ (public) health system, but would not be useful for private billing.
- What about using a model of clearing houses, to offer an incentive for submission? At some moment, funding and/or a financial model for maintaining the databases will be necessary.
- Page 6: LOVD shall be mentioned, as it fulfils the criteria listed in the text.

 

 On information silos:

- It would be good to give a brief description and view point on how silos could be broken down or avoided. What about a model of federated databases?

I confirm that I have read this submission and believe that I have an appropriate level of expertise to confirm that it is of an acceptable scientific standard, however I have significant reservations, as outlined above.

## References

[1] : XLID-causing mutations and associated genes challenged in light of data from large-scale human exome sequencing. *Am J Hum Genet*.2013;93(2) : 10.1016/j.ajhg.2013.06.013 368-83 10.1016/j.ajhg.2013.06.013 23871722PMC3738825

[2] : MYRIAD AFTER MYRIAD: THE PROPRIETARY DATA DILEMMA. *N C J Law Technol*.2014;15(4) :597-637 25544836PMC4275833

# Response

**Published:** 2019-12-02

**Authors:** Caroline Wright

**Affiliations:** University of Exeter, UK

The authors use ‘de-identified’ and ‘pseudonymised’, ‘cryptic link’ and a few other descriptions. It would be good to select the best term or definition and explain it to the readership.

***We have replaced pseudonomised with de-identified throughout and defined it where it first appears as being a process whereby personal identifiers are removed and replaced with linked IDs. We have kept the term cryptic link, as this has a different meaning, but have changed it to “cryptic or hidden link” and explained its purpose e.g. to geographical location.***

 It is unclear what is meant in the abstract with “a small number of …”. Small is hard to define.

***We did not intend to define a number, as it is the combination of a few variants (of variable number) with limited clinical information that together limits the extent to which this information is identifiable.***

****

 “Information robustly linking genetic variants with specific conditions is fundamental biological knowledge.” This is a significant statement that should be explored and explained in more detail, especially if the statement adds that it “should not require consent…”.

***This statement is not intended to apply to personal information but to scientific knowledge in general and is a philosophical assertion. According to the Human Rights Act (see Bartha Knoppers’ work on this) and public health systems such as the NHS in UK, we all have a right to benefit from science. We have slightly amended the sentence to reflect these points.***

 On the recommendations:

 In recommendation 1. The ‘small number’ from the abstract is not well reflected (or vice versa). What are ‘high level’ phenotypes, and why would sharing be limited to these?

***Since very few variants will be causal in any individual, we have not recapitulated the “small number” in the recommendation. The term “high-level” phenotypes is intended to include disease or organ-involvement, and are thus not uniquely identifying even in combination; richer case-level phenotypes may be uniquely identifying, particularly in combination, and therefore may require consent. We have amended the text to clarify this point, though left the term in the recommendation for brevity.***

 Recommendation states no consent in 1. and explicit consent in 3. This dichotomy is not presented in the Abstract. Again, the definition of ‘small’ is crucial, in all instances of policy, defining a ‘cut-off’ is a tricky thing.

***Revised, see above.***

 In general, what about sharing genomic variant that are excluded from disease, i.e. definitely not linked to the disease? The best example is in trans with a known dominant, pathogenic mutation. That information is equally useful.

***We thank the reviewer for this comment, though it raises some difficult questions. Open sharing of such data linked with phenotypes can be confusing. Although there are times when sharing all clinically evaluated variants can be helpful, we feel it is better to focus on a demarcation between disease databases (containing largely pathogenic variants) and population databases (containing largely presumed benign variants).***

 On the Advantages:

 For 2. Clearly, individuals may be identified in data bases by the genotype, for inclusion in clinical trials. With whom shall the data be shared? Companies? What would be the conditions? Who shall be the custodian? How to warrant and permit access? It would be nice to elaborate a bit on this. It is another aspect of variant sharing, that is not covered under the umbrella of variant interpretation.

***We have added a sentence to the paper about this point. We have focused primarily on data sharing with researchers, whether they are clinical, academic or commercial, potentially for any condition.***

 For 3. The moral duty to help has been turned into a legal obligation in France. It may be interesting to cite this, as it is an example or situation that may pop up in other countries.

 For documentation of the situation in France, please visit the following sites:

***We thank the reviewer for this helpful link, and we have added a reference to it into the manuscript.***

 For 4. How shall data be linked to natural history of disease, or vice versa?

***The aim of this article is not to provide details on how data are to be linked, but to provide a conceptual analysis of the issues to facilitate policy in this area. Electronic health records are potentially one method for linking natural history of disease with genetic data, but there are others, and we do not wish to be prescriptive on this point.***

 On the Disadvantages:

 Ref 29 is not tightly linked to the issue of the proposed international sharing of data. Are there other cases/references?

***This reference (now Ref 32) relates to litigation due to variant interpretation and communication, which is directly relevant to data sharing and the point made in this sentence. We are not aware of other better references.***

 There are other, early papers on re-classification, e.g. Piton et al. 2013 ^1^.

***Reference added.***

****

 It is probably worthwhile to mention that some international databases are ‘contaminated’ i.e. contain a wrong and erroneous variant classification. So the curation is essential. Variant database should explicit how the data is collected and managed. In the diagnostic arena, there is a consensus that HGMD data should be explicitly double-checked.

***We have further emphasised this point.***

 The authors also mention the issue of private databases. It is unfortunate indeed that genetic and genomic analyses that are performed in often commercial laboratories do not make it to the public databases. Several large laboratories, mostly in the US, are committed to sharing data, like for instance via ClinVar. However, bad examples do exist as well, and have been denounced early on.

 References could be added, e.g. Conley et al 2014 ^2^.

***Reference added.***

 Also of note is that several databases, that were originally open, have been acquired by commercial companies. The latter has been favourable for their survival, however, the licencing fees are often prohibiting individual laboratories to obtain access. Equally, some companies offer access to their own clinical diagnostic databases, but again, the prices are mostly prohibitive. The data in private databases, especially these that are well curated, may be considered as having a value – as a result of intellectual or other efforts to generate good data – and thus come with a price. How to deal with this?

***We have added a sentence about this point.***

 In parallel, the public laboratories have not been very active in submitting variants. What kind of incentive would be needed to promote data sharing?

***We agree this continues to be an issue and is part of the motivation for writing this article. We acknowledge that public resources are often limited for this sort of activity, and laboratories have historically had variable levels of activity. However, we feel this emphasises the need for better infrastructures to enable fast and efficient data sharing, rather than necessarily creating unrealistic incentives. The motivation to share data already exists, and the majority of regional genetics laboratories in the UK have now submitted variants to a shared database. Moreover, variant sharing is increasingly included in professional best practice guidelines.***

 The statement on informed consent hints at an important shift in the policy of Decipher to request consent. This policy was reportedly very strict in the early days. The position statement pleas for a relaxed (or no) requirement for a written consent. It would be interesting to read how and why the policy has evolved so significantly.

***The field has changed substantially over the last decade as the ubiquity of genetic variation has become more apparent and the rate of diagnoses has increased through large-scale genome-wide sequencing efforts. Decipher continually reviews its data-sharing policy to remain compliant with legal and ethical standards as they evolve and change but there has been no major recent shift in our policy.***

 Finding a balance:

 What is the link between the text and Table 1. Table 1 does not list all the elements that are listed in the text. It would be good to explain to the reader what the aim of Table 1 is.

***Table 1 contains examples of specific data elements for sharing individual genetic variants. We have now explained this more clearly in the text.***

 Open versus controlled access: open access shall best be promoted, given the large number of labs that will either submit or consult.

***We agree.***

****

 Open access databases are not necessarily free. Are there any other incentives to urge (diagnostic or research) laboratories to share variants? The latter are invited to submit in relation to publication, the former? Linking it to reimbursement of the test would be an option for laboratories operating in a ‘fee for service’ (public) health system, but would not be useful for private billing. What about using a model of clearing houses, to offer an incentive for submission? At some moment, funding and/or a financial model for maintaining the databases will be necessary.

***Although we agree with the reviewer on this point, finding incentives for data sharing and solving the long-term funding issues associated with databases is beyond the scope of this paper.***

****

 Page 6: LOVD shall be mentioned, as it fulfils the criteria listed in the text.

***We have added this and a reference.***

 On information silos:

 It would be good to give a brief description and view point on how silos could be broken down or avoided. What about a model of federated databases?

***We have added a point about federated databases and MME to the text.***
